# Work Complexity and Musculoskeletal Symptoms in Healthcare Workers

**DOI:** 10.3390/healthcare14010135

**Published:** 2026-01-05

**Authors:** Elamara Marama de Araujo Vieira, Jonhatan Magno Norte da Silva, Wilza Karla dos Santos Leite, Gilvane de Lima Araújo

**Affiliations:** 1 Postgraduate Program in Physiotherapy, Federal University of Paraíba, João Pessoa 58051-900, PB, Brazil; elamaravieira@gmail.com (E.M.d.A.V.); gilvanearaujofisio@gmail.com (G.d.L.A.); 2 Production Engineering Course, Sertão Campus, Federal University of Alagoas, Delmiro Gouveia 57480-000, AL, Brazil; 3 Physiotherapy Course, Department of Biological and Health Sciences, Federal University of Amapá, Macapá 68902-280, AP, Brazil; wilzakarlas@unifap.br

**Keywords:** pain, occupational health, cluster analysis, workplace, healthcare

## Abstract

**Background/Objectives:** To investigate whether healthcare workers present different characteristics of musculoskeletal symptoms depending on the level of complexity in which these professionals work in the Brazilian Unified Health System. **Methods:** Health professionals were recruited from 24 health institutions, using probabilistic stratified sampling. Data were collected using the Nordic Musculoskeletal Questionnaire. We obtained the questionnaire scores through exploratory factor analysis. Based on the scores, individuals could be grouped into symptom configurations using a non-hierarchical clustering algorithm (K-means). **Results:** The created groups differed in symptom intensity and location but did not differ by level of work complexity, as defined by Brazil’s healthcare sector division. Therefore, regardless of the level of complexity at which professionals perform their activities in the Brazilian Unified Health System, the burden of musculoskeletal symptoms related to the factor under analysis is similar. We developed distinct symptom profiles for each group, accompanied by targeted occupational intervention recommendations. **Conclusions:** This study challenges conventional assumptions by demonstrating that musculoskeletal symptom burden remains consistent across varying levels of work complexity, while providing a practical framework for developing targeted interventions based on symptom profiles.

## 1. Introduction

Musculoskeletal disorders (MSDs) are highly prevalent occupational complaints among healthcare workers worldwide, generally affecting over 70% of these professionals, especially nurses, dentists, and physiotherapists [1]. According to official Brazilian data, in 2024, MSDs accounted for 26.7% (841.8 thousand) of all non-accidental sick leaves in the country [2]. Among these professionals, their estimated frequency is 49.9% [3], and they constitute the second-leading cause of absenteeism [4]. These disorders impair task performance [5] and elevate the risk of diminished work capacity by fivefold [6].

The economic burden of work-related musculoskeletal and connective tissue disorders is substantial. In Japan, these conditions rank as the second-highest driver of presenteeism and medical/pharmaceutical costs, trailing only mental and behavioral disorders. MSDs account for the most significant productivity losses attributable to presenteeism, costing firms $833,327 annually, alongside outpatient medical expenses of $43,935 [7]. Similarly, Chile’s healthcare system incurs annual expenditures of approximately $1.39 billion due to MSDs [8].

From a productivity and health perspective, an individual’s musculoskeletal status predicts functional performance, with effects persisting for six months [9]. Such symptoms incapacitated 21.2% of healthcare workers over 12 months [10]. Furthermore, self-reported MSDs, especially in the upper limbs and spine, correlate with a 27% higher risk of workplace accidents [11].

However, current prevention and mitigation strategies are often inefficient, as interventions tend to be generic and fail to account for workers’ specific contexts or job demands. Although musculoskeletal-related work limitations vary significantly across occupational groups and job demands [12,13], evidence suggests that task interference differs by activity type even when pain thresholds are similar.

In the Brazilian public health system, the work context varies according to demand. This system is structured to stratify healthcare assistance and work by levels of complexity. Consequently, services designated as “primary care” address low-complexity demands, while services designated as “specialized care” handle high-complexity demands. In low-complexity systems, the organization of work addresses disease prevention and screening needs. These services typically consist of small and fixed teams distributed across defined territories, working daily in a predictable flow. In contrast, specialized care comprises outpatient and hospital services that address demands that pose imminent risks to life. These services operate on shift schedules, with significant variability in team composition and the nature of the demands [14].

Therefore, while primary care involves low biomechanical-demand work focused on prevention and health education, specialized care involves patients with life-threatening conditions who often require maneuvers that demand high physical effort from professionals, such as patient mobilization and transfer, cardiopulmonary resuscitation, and basic patient hygiene and feeding care.

Thus, professionals working in these environments address distinct biomechanical demands, with specific organizational structures and work systems. Therefore, it is reasonable to consider that this context may lead these workers to exhibit different symptomatic profiles, which is the hypothesis of analysis in this article. Our aim is therefore to investigate whether healthcare workers exhibit different characteristics of musculoskeletal symptoms depending on the level of complexity of the work they perform in the Brazilian Unified Health System.

Given that (i) occupational interventions (e.g., physical training) significantly reduce pain [15,16,17], yet (ii) guidelines for tailoring such interventions to specific populations, accounting for work and worker heterogeneity, remain unclear [18,19], this study’s findings can guide the development of targeted strategies.

## 2. Methods

### 2.1. Design, Research Field, and Participants

This is an exploratory cross-sectional study and, as such, does not aim to establish causality among the analyzed variables. We investigated workers from 24 health services integrating the Brazilian Unified Health System in municipal management (João Pessoa/Paraíba/Brazil), 20 of which were primary care units, and the others were hospital and specialized emergency care units. We interviewed workers of three hospitals and one emergency care unit.

The sample comprises workers who signed the formal acceptance terms to participate in the research, including those with higher and technical education. In primary care, we considered community healthcare agents (CHAs), professionals who work directly with the user/patient (excluding attendants, administrative assistants, and other support staff). Other inclusion criteria were as follows: workers aged 18 to 65 years, with at least 6 months of experience in the function and the company, who had not been absent from work in the last 3 months, and who worked at least 20 h per week in the function. The exclusion criteria were (1) professionals in a pregnant state; (2) amputee workers, wheelchair users, or workers with chronic mobility limitations; (3) workers diagnosed with genetic diseases affecting mobility.

The participants were informed about the scope of the research and, upon acceptance, instructed to sign the Informed Consent Form. This research followed national and international ethical guidelines, and the authors previously submitted it to the National Research Ethics Committee (CAAE: 79349617.6.0000.5188).

### 2.2. Recruitment

Once available, the list of health care units in the city of João Pessoa, Paraíba, Brazil, was used for recruitment, which consisted of probabilistic sampling (by lottery) of the analysis unit, stratified by type (primary or specialized care). With the units of analysis already established, we developed a list of professionals working in the location. Next, we configure the professional arrangement that could compose the sample in each unit of analysis. In the primary care units, with the limited number of professionals, we included all eligible professionals in the selected units.

**Figure 1:**
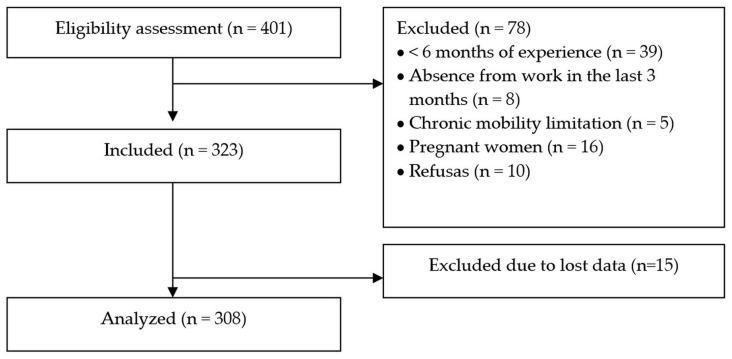
STROBE flow chart.

In contrast, for the specialized care units (hospitals and emergency care units), which had a higher number of professionals and sectors, we first selected the sector to be analyzed by lottery. We then followed the same data-collection procedure used in the primary care units. We repeated this process until the sample size was proportionally reached across primary and specialized care, irrespective of the number of selected analysis units. Figure 1 presents the number of professionals approached for eligibility assessment.

To calculate the sample size, we used the equation from Fávero et al. [20], where z_γ_ = the abscissa of the standard normal distribution for a confidence level γ; $\hat{p}$ = estimated proportion of p; $\hat{q}$ = 1 − $\hat{p}$; N = population size; e = sampling error (maximum allowed difference between p and $\hat{p}$). The calculated sample size was 323 individuals, drawn from an approximate population of 2000 workers, with parameters set at $\hat{p}$ = 50%, e = 5%, an effect size of 1.0, and a 95% confidence level.

(1) $$n=\frac{z_{g}^{2}\;x\;\hat{p}\;x\;\hat{q}\;x\;N}{e^{2}\;\left(N-1\right)+z_{g}^{2}\;x\;\hat{p}\;x\;\hat{q}}$$

### 2.3. Procedures

Data collection was carried out punctually and individually during the work shift in a designated room with appropriate conditions. Before data collection, the research team oriented the workers on the study’s objectives and their participation, in accordance with ethical principles.

**Figure 2:**
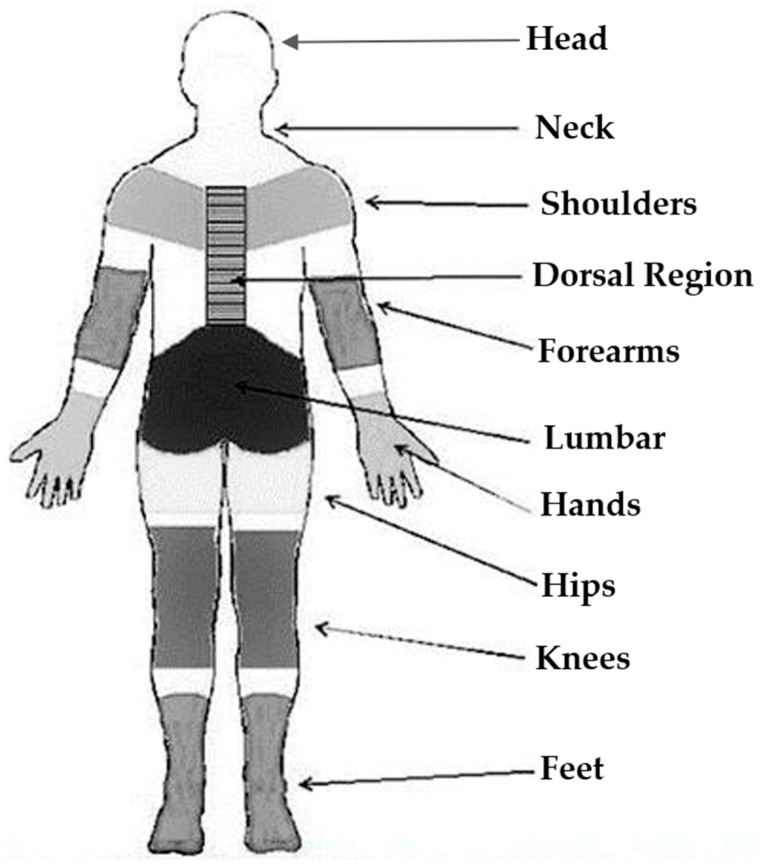
Nordic Musculoskeletal Questionnaire.

We recorded the frequency and intensity of musculoskeletal pain symptoms using the Nordic Musculoskeletal Questionnaire (NMQ), which Pinheiro et al. [21] validated as a morbidity measurement tool (Figure 2). The instrument is a graphical scheme featuring an anatomical body chart, in which respondents assess the frequency and intensity of pain, discomfort, and/or numbness over the preceding 7 days for each body region using a 5-point Likert scale.

The resulting score, ranging from 9 to 45 points, indicates whether symptoms are present. We categorize these scores as low-intensity musculoskeletal complaints (9–18 points), moderate-intensity (19–36 points), and high-intensity (above 36 points) [21]. However, as the questionnaire lacks a single defined threshold, the frequency and intensity of symptoms must be analyzed separately.

### 2.4. Statistical Analyses

The data obtained in the field were initially analyzed descriptively, considering measures of central tendency and normality (Kolmogorov–Smirnov test), followed by hypothesis tests to assess differences between groups (Kruskal–Wallis and Levene Tests, chi-square and Wilcoxon tests).

In this phase, we evaluated the questionnaire’s internal consistency using Cronbach’s alpha. We performed all previously reported tests in R (v. 4.3.1) using the packages psych, clustertend, and dunn.test at the 5% significance level.

Since the NMQ does not provide a score combining symptom presence and intensity, we performed an exploratory factor analysis (EFA) to identify how the questionnaire variables group together and determine which score each factor yields. Subsequently, the factors related to symptomatic characteristics led to the formation of groups to identify a clustering pattern by work complexity level. This approach aimed to determine each group’s specific symptomatology needs.

#### 2.4.1. Factor Analysis

We first assessed the EFA’s adherence to the data and the adequacy of the model assumptions using Bartlett’s test of sphericity, the Kaiser-Meyer-Olkin test (KMO), and the measure of sampling adequacy (MAS) for each variable. Factor extraction used the maximum likelihood method, while the Kaiser criterion (eigenvalues > 1) and the scree plot determined the number of factors. The factor loadings were rotated using the Varimax criterion, thereby making it easier to identify the factor weights for each variable in each factor.

#### 2.4.2. Cluster Analyses

We tested the variables for multicollinearity to certify the technique’s adequacy and employed the K-means algorithm as a similarity measure. To create the groups, we used the EFA factor scores. All factors were tested individually and in combination. After identifying the chosen grouping factor, i.e., the one with the best group-forming indicators, the factorial scores were tested for normality (Kolmogorov–Smirnov test) and for differences in scores across groups (Levene test, Kruskal–Wallis Test, and Dunn’s post hoc test (Holm adjustment).

Effect size estimates, derived using Eta squared (η^2^), are reported alongside the statistical results. Furthermore, confidence intervals (CIs) for the group centroid means were generated via Bootstrap resampling. The number of groups was determined from test and retest, following recommendations by Hair et al. [22]; there is no standard, objective selection procedure. The clustering assessment was performed using the Silhouette coefficient and Davies-Bouldin index.

## 3. Results

The Cronbach’s alpha coefficient of 0.92 (95% CI: 0.91–0.94) indicated acceptable internal consistency for the Nordic Musculoskeletal Questionnaire (NMQ), supporting the validity of the questionnaire data. The study recruited 323 healthcare workers, with equal representation (50%) from primary and specialized care settings in João Pessoa, Paraíba. After excluding 15 questionnaires (4% attrition) due to extensive missing data that precluded score calculation, the final analytical sample comprised 308 workers (162 from primary care).

Table 1 presents the sample’s sociodemographic, lifestyle, and occupational characteristics. The sample characteristics included a mean age of ≥40 years and a predominance of participants with children.

### 3.1. Musculoskeletal Symptom Scores

The dataset, comprising 308 participants and 18 variables (assessing frequency and intensity of symptoms across nine body regions), demonstrated appropriate suitability for exploratory factor analysis (EFA). Bartlett’s test of sphericity (*p* < 0.001) and a Kaiser-Meyer-Olkin (KMO) measure of 0.79 confirmed the data’s adequacy for factor analysis. All body regions showed measure of sampling adequacy (MSA) values between 0.72 and 0.83, indicating strong potential for a factor structure. Additionally, 75% of inter-variable correlation coefficients exceeded 0.30, further supporting the appropriateness of the data for EFA.

**Figure 3:**
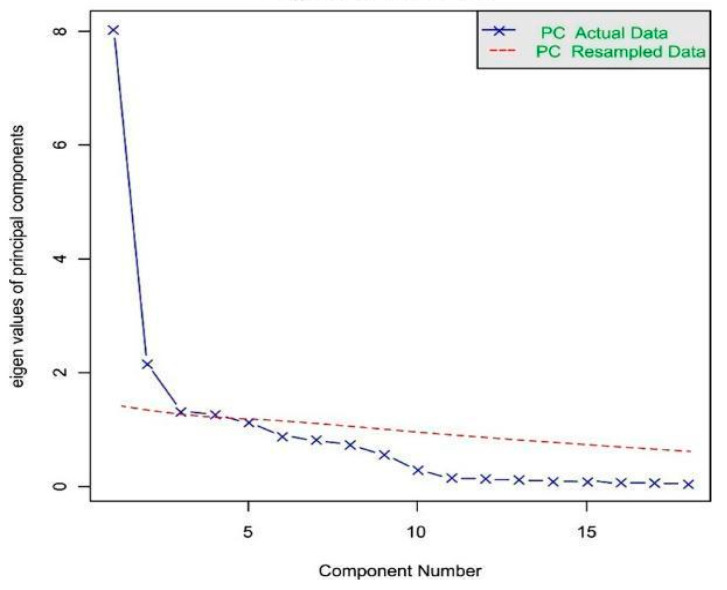
Scree plot for determining the number of factors.

Application of the Kaiser criterion (eigenvalues > 1) yielded a 5-factor solution accounting for 68% of the cumulative variance, exceeding the 60% threshold recommended by Hair et al. [22]. The scree plot (Figure 3) provided supplementary evidence supporting the 5-factor solution.

Factor 1 comprised symptoms associated with the thigh region, Factor 2 with the feet region, and Factor 4 with the knees region. Factor 3 combined symptoms from anatomically proximate regions—wrists, hands, and forearms—where pain frequency and intensity are clinically linked to repetitive movements and sustained muscular tension. Notably, Factor 5 incorporated the most original variables, all about symptom frequency and intensity along the body’s central axis: head, neck, upper back (thoracic region), and lumbar spine. We subsequently used the factor scores from this analysis to cluster individuals with similar symptom profiles (Table 2).

### 3.2. The Cluster Analysis

To form the groups, we adopted the factorial scores generated in factor 5 (axial skeleton). The tests with the other factors and combinations showed unsatisfactory indicators. Although factor 1 has the highest eigenvalue in the analysis, it did not generate distinct groups, possibly because symptoms in this body area are uniform across the sample in terms of frequency and intensity. The use of factor 5 is consistent with the literature, as exemplified by the publications of Jacquier-Bret and Gorce [1], Bucher [23], and Moreira [24], which identify these areas as the most significant for the symptomatic profile of healthcare professionals.

The factorial scores from factor 5 were tested for normality (Kolmogorov–Smirnov test), and with a *p*-value of 0.001, indicating that the data do not follow a normal distribution. The analysis identified three groups (G1 = 96, G2 = 102, G3 = 110) with approximately similar sizes. According to Fávero et al. [20], such balanced group sizes indicate good homogeneity. Furthermore, a sum of squares value of 90.7% demonstrates a good model fit, approaching the maximum possible value of 100%.

The centroids were equal to 1.14 (1.06–1.21, CI 95%), 0.12 (0.06–0.16, CI 95%), and −1.10 (−1.14–−1.05, CI 95%) for G1, G2, and G3, respectively. G1 included factor scores of individuals who had a higher symptom load compared to the mean (evidenced by a positive value greater than 1); G2 included individuals who had a symptom load approximately within the sample mean (value close to zero); and G3 included individuals who had a lower symptom load than the sample mean (denoted by a negative value).

This study tested the difference in factor analysis scores between the groups using the Levene and Kruskal–Wallis tests, which yielded *p*-values of 0.011 (η^2^ = 0.0286) and 0.000 (η^2^ = 0.8872), respectively. The assumption of homogeneity of variances was not met, as assessed by Levene’s test. The Kruskal–Wallis test revealed a statistically significant difference in scores across the three groups.

Post hoc comparisons using Dunn’s test (adjusted *p*-values < 0.001) confirmed that all group pairs differed significantly. The silhouette coefficient of 0.6539 and Davies-Bouldin index of 1.3232 suggest that the objects are well grouped. These results confirm that the clustering procedure successfully created distinct groups with internal homogeneity and external heterogeneity.

Job tasks differ across healthcare tiers due to distinct sector-specific activities. While primary care involves tasks with lower biomechanical overload, time pressure, and multitasking demands, specialized care comprises professionals performing invasive procedures under constant pressure to make life-critical decisions. It is therefore reasonable to assume that symptom patterns likely differ across teams.

To investigate whether any of the groups developed at this stage has a higher prevalence of professionals from a given level of work complexity, we performed z-tests for proportions. In all cases with *p*-values greater than 0.05, the null hypothesis that the proportions are similar was accepted, with no prevalence of any level of work complexity among the symptomatology groups. Regardless of the level of work complexity in which the professional performs their activities in the Brazilian Unified Health System, the load of musculoskeletal symptoms related to the factor under analysis may be similar.

Moreover, we tested the hypothesis of intergroup differences regarding the sociodemographic variables presented in Table 1. In all cases, the results were non-significant (*p*-value > 0.05), minimizing the potential influence of personal factors on the symptomatic characteristics of each formed group.

**Figure 4:**
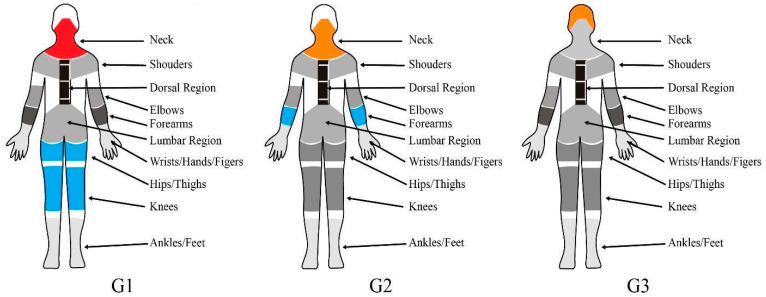
Musculoskeletal symptom profile for each cluster. Legend: Red = Critical area (over half of the sample reported feeling pain frequently or consistently in that region), Orange = Alert area (over half of the sample reported feeling pain sometimes or during the last two weeks in that region); Blue = Stability area (over half of the sample reported feeling pain never or rarely in that region).

Figure 4 presents a symptom profile for each cluster. It is possible to observe that the groups differ in the symptomatic area of highest relevance, with a gradation in the prominence of these symptoms. G1 predominantly exhibits high-frequency symptoms in the cervical and upper back regions, with over 50% reporting frequent or constant pain. In contrast, G2 and G3 report medium- and low-frequency symptoms, respectively. G1 stands out as the only group where over 50% of participants reported no high-frequency symptoms in the thigh region. This finding indicates better preservation of this area than in other body regions within the group.

## 4. Discussion

We identified distinct musculoskeletal symptom profiles among healthcare professionals. The frequency and intensity of symptoms in the axial body region differentiated the groups. However, prior analyses did not reveal a statistically significant association between these profiles and the level of work complexity at which professionals perform their duties (based on Brazil’s healthcare sector division).

The clustering based on symptoms in the axial body region (Factor 5) is consistent with the literature, as the prevalence of musculoskeletal symptoms in the low back and cervical spine is highest among health workers [25,26,27]. It results in losses in the worker’s quality of life [28,29]. It arises from work dynamics, including procedures such as bending several times a day to perform activities and weight-bearing when operating on dependent patients [30,31].

Pain in the upper back, neck, and trapezius is the most critical variable for factor 5 (Factorial loading = 91%). A previous study conducted with Malaysian nurses found that the neck was the region most affected by work-related musculoskeletal disorders, and that among nurses who perceived high physical demands at work, the risk of disorders in this region ranged from 1.68 to 1.83 [32].

It may be related to the variety of tasks performed by workers that require excessive muscular effort and inappropriate postures [12], for example, a large number of functions performed at workbenches or tables that involve neck flexion or a forward head tilt [33]. Other factors such as working time in the sector (>10 years), age (≥50 years) [34], hospital level (tertiary care hospital), work regime (temporary), workload (>45 h), ergonomic factors (bending the neck forward and twisting the neck for long periods), and computer-related factors (prolonged time using the computer daily, the keyboard too close to the edge of the desk) also increase the risk [31,35,36].

The grouping based on axial skeletal symptoms provides an essential picture of the situation, with consistent theoretical support in the literature. However, one cannot fail to consider the other symptoms associated with the factors that did not generate groupings, since they can, in a practical context, substantially aggravate the worker’s overall pain and interfere with their work activities.

Furthermore, these groupings provide a snapshot of the moment. Without preventive and protective measures, occupational exposure from daily work stresses, physically demanding tasks, and the natural aging process will likely aggravate these symptoms over time.

It is noteworthy that in none of the groups were there differences in the frequency of any professional across the level of work complexity (based on Brazil’s healthcare sector division). The homogeneous grouping characteristics across all assessed health professionals reinforce the method’s appropriateness, as these traits reflect a common condition in the health sector.

Recent articles with workers in the manufacturing industry, in which the nature of the work is repetitive and monotonous, showed that musculoskeletal symptoms have a cumulative behavior, i.e., a pain symptom starts in a particular region and expands to others due to the biomechanical compensation process, generating the same pattern of symptom evolution for most workers [37,38].

However, this research indicates that, for healthcare professionals, the behavior of musculoskeletal symptoms is diverse, not merely cumulative, and instead forms distinct profiles characterized by differences in frequency, intensity, and bodily location. In this sample, however, these profiles were not dependent on the work complexity level based on Brazil’s healthcare sector division, nor on the sociodemographic and occupational factors considered in this analysis, suggesting a more complex interaction between the individual and their work beyond these factors.

Studies show that healthcare professionals exposed to more complex, physically demanding tasks or higher workloads exhibit a higher prevalence and intensity of musculoskeletal symptoms [1,39]. However, this study compares not professions, but sectors of practice. This approach disregards the specific particularities of each profession, focusing instead on the sector as a whole, which may have influenced these analytical results.

Furthermore, factors such as occupational stress, work overload, lack of task control, and insufficient social support also contribute to the onset and worsening of musculoskeletal symptoms. Professionals subjected to high psychological demands and low autonomy present more symptoms, regardless of their specific role or sector [1,40,41].

These factors associated with organizational issues, such as ethical conflicts and problematic work relationships, increase the risk of musculoskeletal disorders, particularly in the axial region [42,43]. Personal and psychosocial risk factors, such as age, weight, stress level, skill development, and emotional demands, were significantly associated with musculoskeletal symptoms among Brazilian healthcare workers and are not exclusive to any one sector [44].

Furthermore, the Brazilian public health system has faced shortages and an uneven distribution of professionals for decades, leading to overcrowding and overload, particularly in more peripheral locations [45], a characteristic of some of the healthcare units included in this sample. All these factors may have homogenized the sample, rendering sector-based stratification less influential, as complex psychosocial and organizational risk factors may be affecting all these professionals equally.

Finally, we demonstrate that musculoskeletal symptoms vary across established group profiles, but the work-complexity levels structured by the Brazilian public healthcare system do not directly influence them. This framework represents one component of the contextual factor for these workers, identified by Hünefeld and Meyer [46] as one among several contributors to the configuration of work intensity.

Consequently, although this study considered individual and occupational factors, the complex interaction among personal characteristics, work demands, and context as potential causes or aggravators of musculoskeletal symptoms warrants further investigation.

### 4.1. Strengths

Among the strengths of this study is its innovative approach, as, to our knowledge, no other studies have examined profiles of musculoskeletal disorders by framing the analysis through the lens of work complexity.

Furthermore, the analyses were conducted with a large sample size across multiple analysis units, a sampling method that, according to Ahmed [47], ensures the representativeness of unmodeled establishments and individuals. This leads to more precise estimates and enhances the generalizability of the results to the target population. Additionally, the application of robust statistical methods strengthens the research validity.

### 4.2. Practical Applications and Future Lines of Research

For this sample and others with similar characteristics, devising distinct strategies based solely on the healthcare sector may be misguided. Workers with high symptom frequency may benefit from more intensive, longer-duration interventions. In contrast, those with moderate to low symptom frequency may benefit from more sporadic, monitoring-based interventions. Postural awareness training could prevent or alleviate such symptoms.

In individuals with reduced symptomatic frequency and intensity, the approach should address both symptom prevention and reduction. In G3, the conduct should be directed towards global prevention, since this group does not present prevalent areas with persistent symptoms. However, there is an alert level for the head region, which may indicate problems related to mental workload and tension headaches, requiring specific procedures for the work environment and organization.

This targeted strategy can optimize costs and efforts for occupational health services. Occupational health systems can use the model proposed in this article to screen for risk profiles and schedule personalized interventions centered on workers’ needs. Therefore, repeating the clustering procedure could be an effective strategy for the occupational health and safety sector, though not mandatory, given that our study proposes the primary focus for intervention.

Interventions for these workers should prioritize stretching, strengthening, and relaxation of the cervical and upper-limb musculature, with explicit targeting of the trapezius and scalenes. Additionally, implementing ergonomic workstations adaptable to user anthropometry, along with internal policies fostering autonomy, flexibility, and safe hierarchical relationships, can reduce physical and psychological strain, thereby minimizing harm to the musculoskeletal system.

We encourage replication in other contexts to determine whether similar results are observed across different populations, identify potential distinctions by geographical region or profession, and identify which factors may differentiate the profiles, given their homogeneity across groups in this study.

### 4.3. Limitations

The study’s primary limitation stems from participants’ concurrent employment across multiple healthcare institutions (both public and private), which may lead to work overload and homogenize symptom presentation across hierarchical levels. Furthermore, we did not account for potential confounding variables, such as household responsibilities, sports participation, leisure activities, or psychosocial differences, which might influence symptom patterns and illness manifestation.

Furthermore, the lack of association between symptomatic profiles and occupational and demographic variables limits the practical applicability of the study, hindering the direct replication of interventions for specific worker characteristics.

Furthermore, workers with severe conditions are often on leave, which may introduce a selection bias in this sample. This potential bias, however, is mitigated by the fact that symptom progression occurs on a continuum before leave. Therefore, even if a severely symptomatic individual is absent from a given sector, other workers exposed to the same demands, who are at different stages of symptom progression, would still represent potential inter-sector differences.

An additional methodological limitation involves the exclusive reliance on questionnaire data, which may introduce bias due to variations in respondents’ expectations, prior experiences, or personality traits. However, it is important to acknowledge that these self-reported measures remain essential for capturing workers’ subjective experiences of their work environment—nuances that would be difficult to assess through mechanical means alone. That said, even device-based measurements could introduce similar biases for analogous reasons.

## 5. Conclusions

The objective of this study was to investigate whether healthcare workers exhibit different characteristics of musculoskeletal symptoms depending on the level of complexity of their work in the Brazilian Unified Health System. We identified distinct profiles of musculoskeletal disorders among healthcare workers, particularly concentrated along the central body axis (head, neck, upper back, and lumbar spine).

The main finding indicates that, while the work complexity level (based on Brazil’s healthcare sector division) may not directly determine the symptomatic configuration, professionals within the same sector can still exhibit distinct symptom profiles in terms of location, frequency, and intensity.

One of this study’s key theoretical contributions is identifying the body regions that most significantly impact healthcare professionals’ physical health. The identified symptomatic profiles provide a basis for practical recommendations. For Groups 1 and 2, which exhibit moderate to high symptom intensity and frequency concentrated in the cervical and back regions, an interventional approach is necessary, with greater attention given to Group 1. In contrast, Group 3, which presented low overall symptom frequency and intensity but a distinct concentration of symptoms in the head region, would benefit from a preventive approach with specific attention to mental workload and organizational factors.

## Figures and Tables

**Table 1:** Sample characteristics.

	Primary Care			Specialized Care			
Variables	Mean	±SD	%	Mean	±SD	%	*p*-Value
Age	41.5	11.8		39.6	10.7		0.177 *
BMI	26.4	04.8		26.1	05.4		0.148 *
Profession time	14.2	10.5		13.4	09.4		0.723 *
Working time in the sector	08.1	06.5		06.8	06.5		0.056 *
Gender (Female)			86.4			84.2	0.834 **
Profession							<0.001 **
Physician			12.3			13.7	
Nurse			12.9			28.1	
Physiotherapist			03.7			11.6	
Nursing technician			13.5			18.5	
Dentist			13.5			00.0	
Case-worker			02.4			08.9	
CHA			28.3			00.0	
Others ^a^			13.4			19.2	
Educational level							
High school			08.6			00.0	0.003 **
Technical education			20.9			13.7	
Graduation			26.5			31.5	
Residence/Specialization			42.5			49.3	
Others ^b^			01.2			05.4	
Marital status (Married)			47.5			49.3	0.967 **
Children			62.9			63.7	0.999 **
Smoking			04.3			03.4	0.750
Non-consumers of alcohol			40.1			69.1	<0.001 **
Physical activity practice							
≥30 min per week			45.6			51.3	0.328 **
Weekly workload							<0.001 *
20 h			00.0			15.9	
30 h			01.2			46.7	
40 h			93.8			18.7	
Others ^c^			5.00			18.7	
Contractual relationship							
Service provider			46.9			61.6	0.012 **

Legend: CHA = Community healthcare agents; * Wilcoxon test; ** Chi-square test; Bold = significant *p*-values; ^a^ = Pharmacists, speech therapists, nutritionists, occupational therapists, physical education teachers, and psychologists; ^b^ = Elementary education, master’s degree, doctorate, and post-doctorate; ^c^ = Split work hours related to specific work schedules for the profession or shift work contracts. Example: 1 weekly 12-h shift.

**Table 2:** Factor loading matrix with rotation by the Varimax criterion.

Variables	Factor 1	Factor 2	Factor 3	Factor 4	Factor 5	Commonality
Head (f)	0.08	0.12	0.20	0.15	**0.36**	0.213
Neck (f)	0.08	0.08	0.23	0.14	**0.71**	**0.589**
Dorsal Region (f)	0.15	0.13	0.15	0.03	**0.91**	**0.881**
Forearms (f)	0.19	0.16	**0.88**	0.11	0.33	**0.967**
Lumbar (f)	0.29	0.08	0.22	0.23	**0.50**	0.442
Hands (f)	0.25	0.28	**0.41**	0.20	0.35	0.469
Hips (f)	**0.79**	0.18	0.26	0.24	0.20	**0.822**
Knees (f)	0.19	0.19	0.15	**0.86**	0.16	**0.871**
Feet (f)	0.17	**0.83**	0.19	0.22	0.17	**0.829**
Head (i)	0.11	0.12	0.16	0.08	**0.36**	0.189
Neck (i)	0.05	0.08	0.17	0.11	**0.70**	**0.535**
Dorsal Region (i)	0.12	0.09	0.09	0.01	**0.90**	**0.845**
Forearms (i)	0.22	0.18	**0.79**	0.11	0.31	**0.804**
Lumbar (i)	0.31	0.18	0.18	0.23	**0.48**	0.446
Hands (i)	0.25	**0.33**	**0.34**	0.18	0.28	0.402
Hips (i)	**0.92**	0.18	0.18	0.20	0.20	**0.995**
Knees (i)	0.24	0.25	0.11	**0.88**	0.18	**0.933**
Feet (i)	0.14	**0.94**	0.15	0.19	0.17	**0.995**

Legend: (f) = frequency; (i) = intensity; bold = higher factor loadings for each factor.

## Data Availability

The data presented in this study are available on request from the corresponding author (the data are not publicly available due to privacy or ethical restrictions).
